# Using Convolutional Neural Networks for the Assessment Research of Mental Health

**DOI:** 10.1155/2022/1636855

**Published:** 2022-05-09

**Authors:** Yanbing Liu

**Affiliations:** School of Management, Northwestern Polytechnical University, Xi'an 710129, Shanxi, China

## Abstract

Existing mental health assessment methods mainly rely on experts' experience, which has subjective bias, so convolutional neural networks are applied to mental health assessment to achieve the fusion of face, voice, and gait. Among them, the OpenPose algorithm is used to extract facial and posture features; openSMILE is used to extract voice features; and attention mechanism is introduced to reasonably allocate the weight values of different modal features. As can be seen, the effective identification and evaluation of 10 indicators such as mental health somatization, depression, and anxiety are realized. Simulation results show that the proposed method can accurately assess mental health. Here, the overall recognition accuracy can reach 77.20%, and the *F*1 value can reach 0.77. Compared with the recognition methods based on face single-mode fusion, face + voice dual-mode fusion, and face + voice + gait multimodal fusion, the recognition accuracy and *F*1 value of proposed method are improved to varying degrees, and the recognition effect is better, which has certain practical application value.

## 1. Introduction

With the development of economy and the acceleration of the pace of life, people's life pressure is becoming bigger and bigger, and mental health problems have become the focus of global attention. At present, the methods of mental health assessment are mainly based on experts' assessment or self-assessment, which is assessed from the perspective of the patient and the practitioner. Briggs Hannah et al. explored the thoughts, feelings, and educational requirements of nursing staff and nurses at the clinical help desk of emergency medical services, and the focus was on the classification tools used for calls and uses related to mental health. Here, quantitative data are analyzed by descriptive statistics, and qualitative data are analyzed by subject analysis. Thus, mental health assessment and triage of patients and their families are realized [1]. Scelzo Anna evaluated mental health in the form of questionnaires and believed that a good mental health assessment is conducive to promoting healthy aging [2]. Michael R. Hass et al. proposed a concept of case conceptualization and realized the assessment of students' mental health by determining students' psychological needs and writing goals. This method is better than the traditional evaluation process [3]. Fortuna Lisa R. adopted the 2.2 pros and cons method to introduce trauma narrative in the process of sheltered mental health assessment, so as to improve the accuracy of mental health assessment [4]. Scott A. Bresler, Ph. D., reviewed the mental health assessment by forensic in the digital era and believed that the rational use of Internet data is conducive to accurately assess mental health [5]. Higuchi Masakazu et al. constructed a mental health assessment system based on voice modes in a mobile device based on voice, which opens the mental health voice assessment with certain foresight [6]. Newson Jennifer J. et al. assessed the Chinese and Canadian interactive mental health by taking a pilot primary care outpatient clinic led by nurse practitioners as the research object, which is conducive to strengthening the mental health communication between clinicians and patients [7]. OReilly Michelle et al. analyzed 28 videos recording British children's psychology by using discourse psychology, established a rhetorical case to prove the clinical need, and believed that children's mental health is related to parents' teaching by words and deeds [8]. Since then, with the development of information technology, people began to introduce computer-aided methods to evaluate psychology, such as Heesacker Martin using computer system, and CNN proposed by some scholars to evaluate psychology [9–11]. The above research indicates that most of the current mental health assessment methods are mainly based on experts' experience and analysis, and there is a certain degree of subjectivity. In order to better objectively assess mental health, an automatic intelligent assessment method of mental health based on the rapidly developing convolutional neural network is proposed.

## 2. Basic Methods

### 2.1. Introduction to OpenPose Algorithm

**Figure 1:**
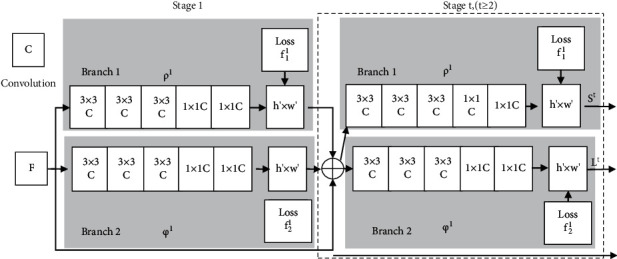
Flow chart of OpenPose algorithm.

OpenPose algorithm is a bottom-up algorithm based on convolutional neural network, which is suitable for single and multiperson pose recognition and has good robustness. The basic structure of OpenPose algorithm is shown in Figure 1, and there are two branches and multistage convolutional neural networks [12]. Here, the yellow and blue parts represent one branch, respectively, and the left and right parts represent two phases, respectively. The yellow branch is used to describe the confidence map of face and posture key points, and the blue branch is used to describe the correlation degree of each key point. The left part is responsible for generating detection confidence maps and partial affinity domains, and the right part is responsible for connecting the prediction results of different branches of yellow and blue to improve the prediction accuracy.

### 2.2. Introduction to Multimodal Fusion

**Figure 2:**
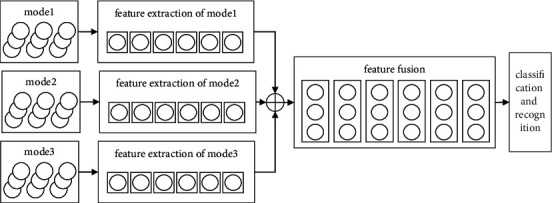
Multimodal fusion and recognition based on feature layer.

Multimodal fusion refers to the fusion of feature information of different modes [13], including three fusion modes, namely, data layer fusion, feature layer fusion, and decision layer fusion. Data layer fusion first combines data, extracts features from the combined data, and then inputs them into a classifier for recognition. Feature layer fusion extracts different modal information data features separately and inputs the combined features into a classifier for recognition. Decision layer fusion extracts data features separately, identifies each extracted feature, and finally fuses the recognition results. In practical applications, multimodal fusion based on data layer plays a positive role in recognition task, but its fusion efficiency is low. The multimodal fusion method based on feature layer may increase the amount and difficulty of calculation because it cannot screen effective features, thus reducing the model recognition results. The fusion method based on decision layer only combines the results of different modes but theoretically does not really integrate the information of all modes [14, 15]. According to the characteristics of mental health assessment mainly from three aspects of human face, voice and gait, as well as reference [16], the multimodal fusion method based on feature layer is adopted, and its basic process is shown in Figure 2.

### 2.3. Introduction to Attention Mechanism

**Figure 3:**
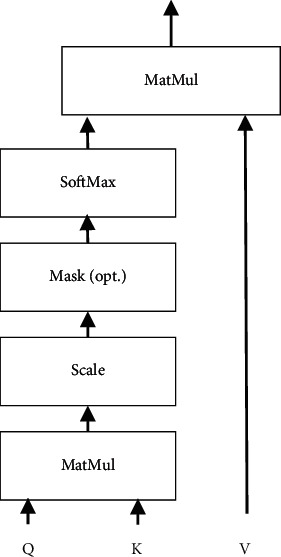
Structure of classical attention mechanism.

Attention mechanism is a kind of perception mode that simulates the human brain to selectively attach importance to useful information and discard useless information, which is first applied in the field of visual images. In recent years, with the in-depth study of attention mechanism, it has been widely used in image recognition, recommendation system, and other fields. The attention mechanism usually follows the form of query (Q), keyword (K), and weight value (V). The structure of the classical attention mechanism is shown in Figure 3 [17].

When the attention mechanism is introduced to distribute weight, formula (1) can be used to distribute weight [18]:

(1) $$\begin{array}{c} \text{Attention}\left(Q,K,V\right)=\sum_{i=1}^{L}\text{Similarity}\left(Q,K_{i}\right)*V_{i}, \end{array}$$

where *L* represents the number of keywords and *Similarity* ( ) means similarity calculation function, which usually includes the following three functions:

(2) $$\begin{array}{c} \text{Double }-\text{linear model Similarity}\left(Q,K_{i}\right)=K_{i}^{T}WQ,\\ \text{Dot product model Similarity}\left(Q,K_{i}\right)=K_{i}^{T}Q,\\ \text{Scale dot product model Similarity}\left(Q,K_{i}\right)=\frac{K_{i}^{T}Q}{\sqrt{\text{d}}}, \end{array}$$

where *W* represents the learnable parameter and *d* represents the dimension of keyword and weight value.

### 2.4. Introduction of SVM

SVM algorithm is a nonstatistical classification algorithm, and its basic principle is based on nonlinear transformation regression function to map sample data to high-dimensional feature space, which can realize the sample data conversion. Its kernel function is defined as follows:

(3) $$\begin{array}{c} K\left(x,z\right)=\varphi {\left(x\right)}^{T}\varphi \left(z\right), \end{array}$$

where *x* and *z* represent data points in the original space and *φ* represents nonlinear transformation. In general, the kernel function of SVM is Gaussian kernel function, as shown in the following:

(4) $$\begin{array}{c} K\left(x,z\right)=\exp\left(-\frac{{\left\|x-z\right\|}^{2}}{2\sigma^{2}}\right), \end{array}$$

where *z* represents the center value of Gaussian function and *σ* represents the width parameter of function.

## 3. Using Convolutional Neural Networks for the Assessment Research of Mental Health

### 3.1. Indicators of Mental Health

In this experiment, referring to literature, there are 10 indicators for mental health, which are shown in Table 1 [19].

### 3.2. Overall Framework of the Model

**Figure 4:**
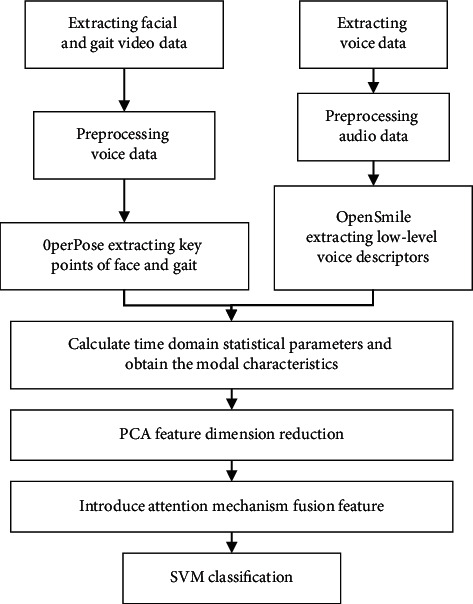
Structure of multimodal fusion mental health assessment model with attention mechanism.

Based on the above analysis, multiple modes of face, voice, and gait are integrated based on the OpenPose algorithm of convolutional neural network, and attention mechanism is used to allocate the weight of different modes reasonably. A mental health assessment model of multimode fusion with the introduction of the attention mechanism is proposed, and its overall framework is shown in Figure 4. Firstly, OpenPose algorithm is used to extract key points of human face and posture, and openSMILE is used to extract low-level voice descriptors. Then, the modal characteristics can be calculated by time domain statistical parameters. Finally, attention mechanism is introduced to allocate the weight of each mode reasonably, and support vector machine (SVM) is used to classify and recognize the mental health assessment.

#### 3.2.1. Feature Extraction

For feature extraction of face and gait images, OpenPose algorithm is adopted to extract key points of face and gait. At the same time, face data and gait data are input into the algorithm to generate detection confidence graph combination *S* and confidence graph unit *S*_*j*_, whose calculation formulas are shown as follows:

(5) $$\begin{array}{c} S=\left(S_{1},S_{2},\ldots ,S_{j}\right),\;j\in \left\{1,\ldots ,J\right\}, \end{array}$$

(6) $$\begin{array}{c} S_{j}=i^{w\times h}, \end{array}$$

where *J* values are 68 and 18, respectively; then coordinate set *Fcoo*_*t*_ of key points of face image in frame *T* and coordinate set *Gcoo*_*t*_ of key points of gait image in frame *T* can be expressed as follows:

(7) $$\begin{array}{c} {\text{Fcoo}}_{t}=\left\{\left(x_{1},y_{1}\right),\left(x_{2},y_{2}\right),\ldots ,\left(x_{68},y_{68}\right)\right\}, \end{array}$$

(8) $$\begin{array}{c} {\text{Gcoo}}_{t}=\left\{\left(x_{1},y_{1}\right),\left(x_{2},y_{2}\right),\ldots ,\left(x_{18},y_{18}\right)\right\}. \end{array}$$

Here, openSMILE method is used to extract the short time energy, formant, pitch frequency, and MFCC of voice features. The short-time energy *E*(*i*) of frame *i* voice signal *y*_*i*_*n* can be calculated by formula (11):

(9) $$\begin{array}{c} E\left(i\right)=\sum_{n=0}^{L-1}y_{i}^{2}n,\;1\leq i\leq f_{n}. \end{array}$$

By calculating data(*n*) of voice signal and carrying out Fourier transform, pitch frequency PF is solved.

(10) $$\begin{array}{c} \text{data}\left(n\right)=u\left(n\right)\times v\left(n\right), \end{array}$$

where *v*(*n*) represents the corresponding filtering of sound channel and *u*(*n*)*u(n)* represents the excitation response of glottis pulse.

The formant parameters For1, For2, and For3 of voice signal peak are calculated by LPC root method [20].

According to formula (11), the spectrum of voice signal is calculated. Combined with Mayer filter and discrete cosine transform, the 12th-order MFCC features are obtained, which can be expressed as formula (12):

(11) $$\begin{array}{c} P=\frac{{\left|\text{FFT}\left(x_{i}\right)\right|}^{2}}{N}, \end{array}$$

(12) $$\begin{array}{c} \text{MFCC}=\left\{{\text{mfcc}}_{1},{\text{mfcc}}_{2},\ldots ,{\text{mfcc}}_{12}\right\}, \end{array}$$

where *P* is the power spectrum; FFT is the fast Fourier transform; *X*_*i*_ is the voice signal; and *N* = 512.

#### 3.2.2. Calculating Time Domain Statistical Parameter

Time domain features can describe the characteristics of different data in time dimension. In addition, arithmetic sum, mean, minimum, maximum, variance, standard deviation, skewness, kurtosis, and correlation coefficient between two axes are selected as the calculation types of time domain feature statistical parameters by referring to literature [21, 22].

#### 3.2.3. Introducing Attention Mechanism

Similarity calculation of mental health assessment query and keyword with attention mechanism includes two aspects, namely, dot product calculation of vector check and cosine similarity calculation, as shown in the following formulas [23]:

(13) $$\begin{array}{c} \text{similarity}1\left(\text{Query},{\text{Key}}_{i}\right)=\text{Query}•{\text{Key}}_{i}, \end{array}$$

(14) $$\begin{array}{c} \text{similarity}2\left({\text{Query,Key}}_{i}\right)=\frac{\text{Query}•{\text{Key}}_{i}}{\left\|\text{Query}\right\|•\left\|{\text{Key}}_{i}\right\|}. \end{array}$$

Then, the weight coefficient is solved through normalization operation, as shown as follows:

(15) $$\begin{array}{c} a_{i}=\text{soft}\max\left({\text{Sim}}_{i}\right)=\frac{e^{{\text{Sim}}_{i}}}{\sum_{j=1}^{L_{x}}e^{{\text{Sim}}_{i}}}. \end{array}$$

Finally, formula (16) shows the weighted sum of weight coefficients, the final fusion feature Fusatt can be obtained [24]. The size of Fusatt is 103*∗*4, and its calculation method is shown in formula (17) [25]:

(16) $$\begin{array}{c} \text{Attention}\left(\text{Query,Source}\right)=\sum_{i=1}^{L_{x}}a_{i}•{\text{Value}}_{i}, \end{array}$$

(17) $$\begin{array}{c} {\text{Fus}}_{\text{att}}=\left[w1\times F_{\text{fea}},w2\times V_{\text{fea}},w3\times G_{\text{fea}}\right]. \end{array}$$

In formula (17), *F*_fea_, *V*_fea_,  and*G*_fea_ represent facial, phonetic, and gait features, respectively.

#### 3.2.4. SVM Classification

According to the evaluation indicators of mental health, there are 10 SVM classifiers trained to judge different mental health conditions, corresponding to 10 mental health indicators such as somatization, depression, and anxiety. Each psychological index includes negative and positive two states, corresponding to not suffering from or suffering from the corresponding psychological disease of this index.

## 4. Simulation Experiment

### 4.1. Construction of Experimental Environment

This experiment is conducted on Windows7 operating system with Intel Xeon Silver4110 CPU; graphics card is NVIDIA GeForce GTX 1080Ti with of 16 G memory; the memory is 128 G; and the development language is Python.

### 4.2. Data Sources and Preprocessing

#### 4.2.1. Data Sources

In this experiment, facial, gait, and voice data of 680 employees in a company collected by Guangdong Electric Power Research Institute and mental health data collected in the form of questionnaires are selected as experimental data. The basic information is shown in Table 2 [26].

#### 4.2.2. Data Preprocessing

To avoid the impact of invalid data on model performance, this experiment deletes and processes the invalid data of some missing values contained in the data set and finally obtains 672 valid data samples. In addition, considering that there may be noise and background sound in the acquisition process of facial, voice, and gait data, the video and audio data are preprocessed, respectively.

For the video data, GaussianBlur function is called to denoise the video data, and then short time series video is generated by resampling, so as to improve the proportion of effective information. For facial video data, a video with a duration of 30 s is used as a segment; for gait video data, a video with a duration of 8 s is taken as a segment [27, 28]. The preprocessed face and gait are *F* and *G*, respectively, so the video data can be expressed as

(18) $$\begin{array}{c} F=\left\{f_{1},f_{2},\ldots ,f_{t}\right\},\\ G=\left\{g_{1},g_{2},\ldots ,g_{t}\right\}, \end{array}$$

where *t* is determined by the size of video frames and *f*_*t*_ and *g*_*t*_ are single-frame facial and gait images.

For audio data, the first is to delete incomplete recording data; the second is to call wiener filtering method in Wiener function to denoise data; thus the random noise in audio is eliminated; finally, the voice is divided into multiple sequence combinations within 1 s to obtain audio set *V*, which can be expressed as

(19) $$\begin{array}{c} V=\left\{v_{1},v_{2},\ldots ,v_{t}\right\}, \end{array}$$

where *t* is determined by the length of audio and voice; *v*_*t*_ is the data storage format; and the storage format of *v*_*t*_ in this experiment is matrix storage.

Through the above preprocessing, a total of 658 valid data samples are obtained in this experiment.

### 4.3. Parameter Settings

In this experiment, parameters of SVM are set as follows: the kernel function is Gaussian function; degree = 3; and the penalty coefficient of error term is 1.

### 4.4. Evaluation Indicators

Accuracy, recall, precise, and *F*1 values are usually selected as indicators for model performance evaluation. The calculation methods are as follows:

(20) $$\begin{array}{c} \text{accuracy}=\frac{\text{TP}+\text{TN}}{\text{TP}+\text{TN}+\text{FP}+\text{FN}},\\ \text{recall}=\frac{\text{TP}}{\text{TP}+\text{FN}},\\ \text{presion}=\frac{\text{TP}}{\text{TP}+\text{FP}},\\ F1=2\times \frac{\text{precise}\times \text{recall}}{\text{precise}+\text{recall}}, \end{array}$$

where TP and TN represent true positive cases and true negative cases and FP and FN represent false positive cases and false negative cases. According to the formulas, the higher the accuracy, accuracy, and recall, the better the model performance.

However, the accuracy and recall cannot grow at the same time. To balance the two, *F*1 value index is proposed. The higher *F*1 value indicates that the accuracy and recall are most balanced. Based on the above analysis, accuracy and *F*1 values are finally selected as indicators to evaluate the performance of model.

### 4.5. Experimental Results

#### 4.5.1. Method Verification

**Figure 5:**
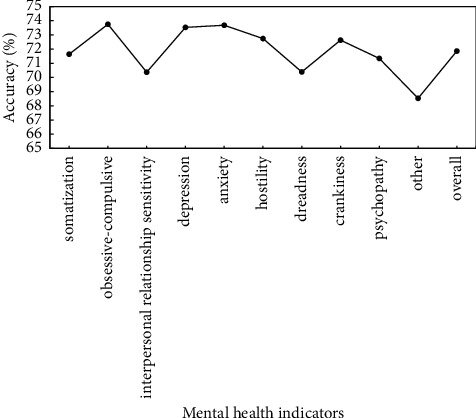
Recognition results based on facial single mode.

To verify the effectiveness of proposed method, the preprocessed data are used to verify the proposed method, and identification accuracy of somatization, depression, anxiety, and other mental health indicators is used as evaluation criteria. Figures 5∼7 show the recognition results based on the single mode of face, voice, and gait; Figures 8∼10 show the recognition results of face + voice, face + gait, and voice + gait; Figure 11 shows the multimode recognition results of face + voice + gait; Figure 12 shows the recognition results of introducing attention mechanism based on Figure 11.

As can be seen from Figure 5, the overall recognition accuracy of recognition method based on facial single mode for all mental health indicators is 71.86%, among which the recognition accuracy of obsessive-compulsive and anxiety indicators is higher, reaching 73.75% and 73.53%, respectively. The recognition accuracy of other indicators is the lowest, reaching 68.53%. In addition, the overall *F*1 value of the method is 0.71.

**Figure 6:**
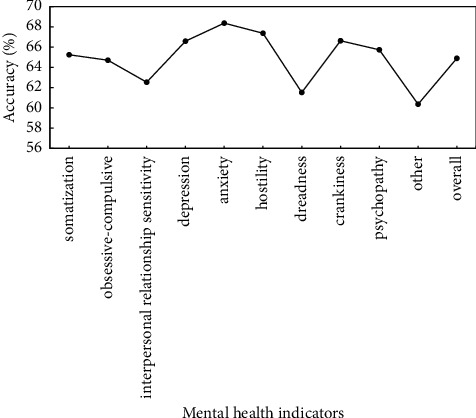
Recognition results based on voice single mode.

As can be seen from Figure 6, the overall recognition accuracy of recognition method based on voice single mode for all mental health indicators is 64.89%. Among them, the recognition accuracy of anxiety and hostility indicators is relatively high, reaching 68.36% and 67.36%, respectively. The recognition accuracy of other and dreadness indicators is relatively low, reaching 60.36% and 61.52%, respectively. In addition, the overall *F*1 value of the method is 0.65.

**Figure 7:**
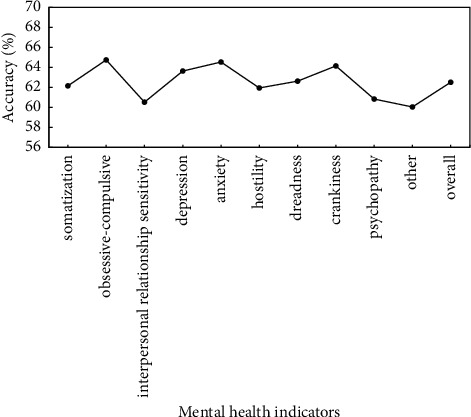
Recognition results based on gait single mode.

As can be seen from Figure 7, the overall recognition accuracy of recognition method based on gait single mode for all mental health indicators is 62.51%. Among them, the recognition accuracy of obsessive-compulsive and anxiety indicators is relatively high, reaching 64.74% and 64.53%, respectively. However, the recognition accuracy of other indicators, interpersonal relationship sensitivity, and psychopathy is relatively low, reaching 60.04%, 60.52%, and 60.83%, respectively. In addition, the overall *F*1 value of the method is 0.60.

**Figure 8:**
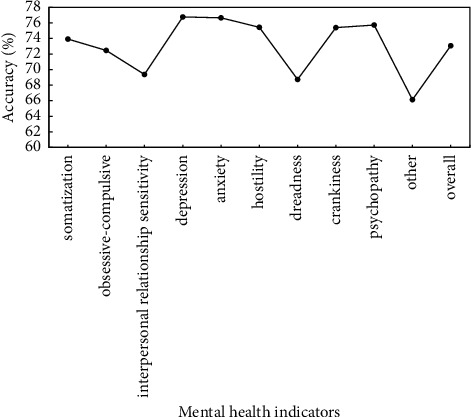
Recognition results based on face + speech dual-modal fusion method.

As can be seen from Figure 8, the recognition accuracy of recognition method based on the face + voice dual-modal fusion for mental health indicators is high, and the overall recognition accuracy is 73.06%. Among them, the recognition accuracy of depression and anxiety indicators is higher, reaching 76.76% and 76.65%, respectively. The recognition accuracy rate of other and dreadness indicators is the lowest, reaching 66.13% and 68.73%, respectively. In addition, the overall *F*1 value of the method is 0.74. Compared with the recognition method based on single mode, the average recognition accuracy is improved by 1.42%, and the average *F*1 value is increased by 11.71%.

**Figure 9:**
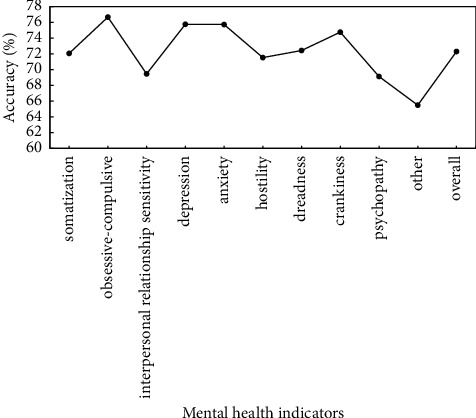
Recognition results based on face + gait dual-modal fusion method.

As can be seen from Figure 9, the recognition accuracy of recognition method based on the face + gait dual-modal fusion for mental health indicators is high, and the overall recognition accuracy is 72.30%. Among them, the recognition accuracy of obsessive-compulsive is high, reaching 76.67%, while the recognition accuracy of other indicators is low, reaching 65.49%. In addition, the overall *F*1 value of the method is 0.71.

**Figure 10:**
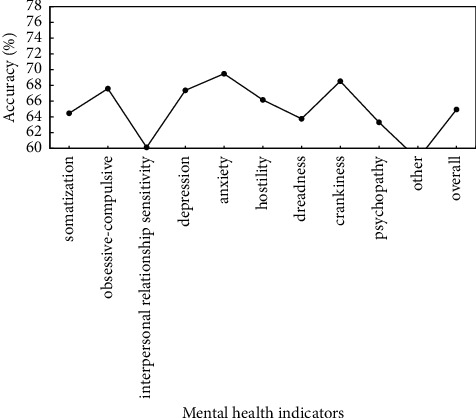
Recognition results based on voice + gait dual-modal fusion method.

As can be seen from Figure 10, the overall recognition accuracy of recognition method based on voice + gait dual-modal fusion for mental health indicators is 64.94%. Among them, the recognition accuracy of anxiety and crankiness is 69.48% and 68.53%, respectively, while the recognition accuracy of other indicators is 58.64%. In addition, the overall *F*1 value of the method is 0.66.

**Figure 11:**
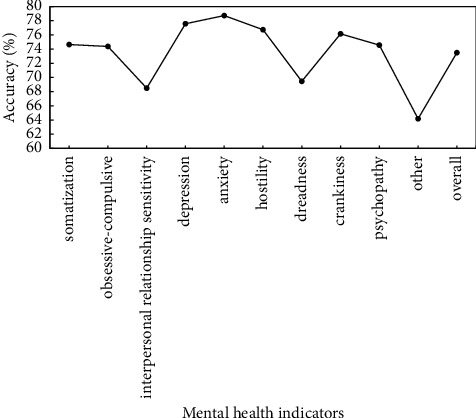
Recognition results based on facial + speech + gait multimodal fusion method.

As can be seen from Figure 11, compared with the recognition methods based on single-modal and multimodal fusion, the overall recognition accuracy of recognition method based on facial + speech + gait multimodal fusion for various mental health indicators is improved to different degrees, reaching 73.49%. Among them, the recognition accuracy of anxiety reaches 78.72%, and the recognition accuracy of other indicators is lower, reaching 64.18%. In addition, the overall *F*1 value of the method is 0.75.

**Figure 12:**
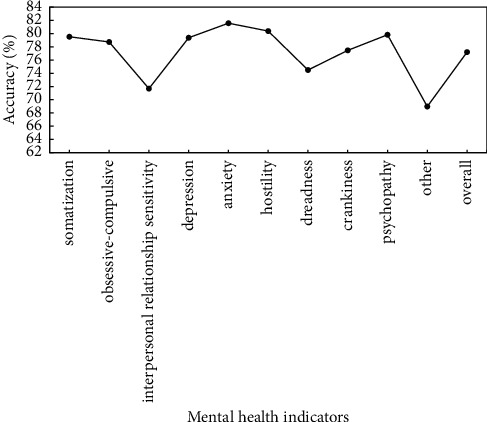
Recognition results of face + speech + gait multimodal fusion method with attention mechanism.

As can be seen from Figure 12, the recognition method of face + voice + gait multimodal fusion with attention mechanism can accurately evaluate mental health. The recognition accuracy of anxiety and hostility can reach more than 80%, and the recognition accuracy of somatization, depression, and psychopathy can reach more than 79.3%. The overall recognition accuracy of mental health indicators is 77.20%. In addition, the overall *F*1 value of the method reaches 0.77.

In conclusion, the proposed method of mental health assessment can effectively improve the recognition accuracy of mental health indicators and *F*1 value by fusing face, voice, and gait. In addition, attention mechanism is introduced. Compared with recognition method based on single-modal, double-modal, and multimodal fusion, the proposed method of mental health assessment has better recognition effect and has certain effectiveness.

#### 4.5.2. Comparison of Methods

**Figure 13:**
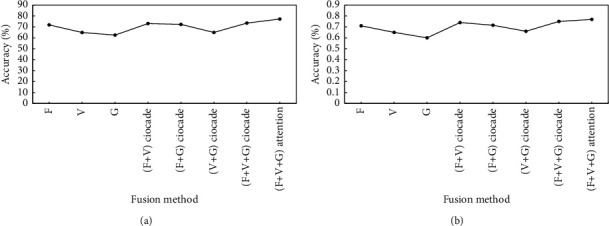
Results of different methods to assess mental health. (a) Comparison of recognition accuracy. (b) Comparison of *F*1 values.

To further verify the effectiveness and superiority of proposed method, the evaluation effect is compared with that of the commonly used mental health assessment method. The recognition accuracy of different methods is shown in Figure 13(a), and the *F*1 value is shown in Figure 13(b). In the figures, *F*, *V*,  and *G* are single-modal recognition methods based on face, voice, and gait, respectively. *F*+*V*, *F* + *G*, and *V*+*G* are dual-modal fusion recognition methods based on face + voice, face + gait, and voice + gait, respectively. *F*+*V*+*G* is face + voice + gait multimodal fusion recognition method. (*F*+*V*+*G*) attention is a multimodal fusion recognition method that introduces attention mechanism. Figure 13(a) shows that the recognition accuracy of multimodal fusion method is higher than that of single-modal and dual-modal fusion recognition methods. The proposed multimodal fusion method with attention mechanism has the highest recognition accuracy, reaching 77.2%. As can be seen from Figure 13(b), the proposed multimodal fusion recognition method based on the attention mechanism has the highest *F*1 value, reaching 0.77. Compared with the recognition method based on face single-modal fusion, *F*1 value is increased by 9.10%. Compared with the recognition method based on dual-modal fusion, the average *F*1 value is improved by 11.53%. Compared with the multimode fusion recognition method without attention mechanism, the *F*1 value is improved by 2.60%. The experimental results show that the proposed method has certain effectiveness and superiority in mental health assessment and solves the problem of insufficient information based on single mode and double mode. Meanwhile, the attention mechanism is introduced to reasonably allocate the weight of face, voice, and gait modes and improve the model performance. Compared with the recognition method based on single-modal fusion and dual-modal fusion, and the multimodal fusion recognition method without attention mechanism, the recognition accuracy and *F*1 value of the proposed method are improved to varying degrees, and the recognition effect is better.

## 5. Conclusion

To sum up, the proposed mental health assessment method based on convolution neural network can realize effective identification and evaluation of somatization, depression, anxiety, and other mental health indicators, where modal characteristics of face, voice, and gait are fused. In addition, attention mechanism is introduced to allocate different modal weights. The overall accuracy can reach 77.20%, and *F*1 value can reach 0.77. Compared with the recognition methods based on face single-modal fusion, face + voice dual-modal fusion, and face + voice + gait multimode fusion, the recognition accuracy and *F*1 value of the proposed method are improved to varying degrees, and the recognition effect is better, which has certain practical application value. However, due to the limitation of conditions, there are still some deficiencies to be improved, mainly focusing on the construction of data set. At present, there are few data sets about mental health in China, and the size of data sets has a great influence on mental health assessment model, so the number of data sets selected in this paper is far from the requirements. Therefore, it is necessary to build a mental health database with large amount of data and high quality. The next step is collect more original data to enhance the model performance and improve the recognition accuracy of model.

## Figures and Tables

**Table 1:** Description of mental health indicators.

Indicators of mental health	Description
Somatization	Somatization is mainly used to express the discomfort of subjects
Obsessive-compulsive	A repeated and persistent compulsion or behavior
Interpersonal relationship sensitivity	A strong sense of inferiority and uneasiness in dealing with others
Depression	Having a lot of pessimism inside and being in a low mood
Anxiety	Showing excessive agitation, restlessness, nervousness, etc.
Hostility	Feelings of hostility or antagonism towards others
Dreadness	An intense and unnecessary fear towards certain objects or situations
Crankiness	Excessive attachment to certain things or ideas
Psychopathy	An emotional, cognitive, or behavioral disorder caused by dysfunctions in the brain
Other	Diet status, sleep quality, and so on

**Table 2:** Basic information of experimental data.

Information	Project	Number
Gender	Male	641
Age	Female	39
	Under 30 years old	114
	31 ∼ 40 years old	146
	41 ∼ 50 years old	226
	Over 51 years of age	194
Education level	Primary school or below	7
	Junior high school	44
	High school or technical secondary school	127
	Junior college	155
	Undergraduate	284
	Master's degree or above	63
Seniority	1 ∼ 5 years	107
	6 ∼ 10 years	96
	10 ∼ 15 years	52
	16 ∼ 20 years	128
	20 ∼ 30 years	288
	More than 30 years	9

## Data Availability

The experimental data used to support the findings of this study are available from the author upon request.
